# Introgression of *tsv1* improves tungro disease resistance of a rice variety BRRI dhan71

**DOI:** 10.1038/s41598-022-23413-4

**Published:** 2022-11-05

**Authors:** Tapas Kumer Hore, Mary Ann Inabangan-asilo, Ratna Wulandari, Mohammad Abdul Latif, Sheikh Arafat Islam Nihad, Jose E. Hernandez, Glenn B. Gregorio, Teresita U. Dalisay, Maria Genaleen Q. Diaz, Balachiranjeevi Ch., B. P. Mallikarjuna Swamy

**Affiliations:** 1 International Rice Research Institute (IRRI), DAPO Box 7777, Metro Manila, Philippines; 2 University of the Philippines Los Banos (UPLB), College, Laguna, Philippines; 3 Bangladesh Rice Research Institute (BRRI), Gazipur, Bangladesh; 4 The South East Asian Regional Center for Graduate Study and Research in Agriculture (SEARCA), Los Banos, Philippines

**Keywords:** Biotechnology, Plant sciences

## Abstract

Rice Tungro disease poses a threat to rice production in Asia. Marker assisted backcross breeding is the most feasible approach to address the tungro disease. We targeted to introgress tungro resistance locus *tsv1* from Matatag 1 into a popular but tungro susceptible rice variety of Bangladesh, BRRI dhan71. The *tsv1* locus was traced using two tightly linked markers RM336 and RM21801, and background genotyping was carried out using 7 K SNPs. A series of three back crosses followed by selfing resulted in identification of plants similar to BRRI dhan71. The background recovery varied at 91–95% with most of the lines having 95%. The disease screening of the lines showed moderate to high level of tungro resistance with a disease index score of ≤ 5. Introgression Lines (ILs) had medium slender grain type, and head rice recovery (59.2%), amylose content (20.1%), gel consistency (40.1 mm) and gelatinization temperature were within the acceptable range. AMMI and Kang’s stability analysis based on multi-location data revealed that multiple selected ILs outperformed BRRI dhan71 across the locations. IR144480-2-2-5, IR144483-1-2-4, IR144484-1-2-2 and IR144484-1-2-5 are the most promising lines. These lines will be further evaluated and nominated for varietal testing in Bangladesh.

## Introduction

Rice provides daily calories and nutrition to the majority of the Asian population, and it is becoming a popular diet in Africa and Latin America as well^1^. The demand for rice is increasing globally, so there is a dire need to increase rice production and productivity. As per some estimates, we need to produce additional 10 million tons of rice every year to feed the increasing population^2–6^. However, rice production is constrained by several major diseases such as bacterial blight, blast, sheath blight, and rice tungro^7–9^. Among the more than 17 viral diseases of rice, rice tungro (RT) incidence and severity are high in Asia^10^. Annually 10% ($1.5 billion) of rice yield losses are attributed to RT, and there have been several incidences of complete crop failures in South and South East Asian countries^11–13^.

RT disease is caused by two viral particles, namely rice tungro spherical virus (RTSV, an RNA virus) and rice tungro bacilliform virus (RTBV, a DNA virus). Both are easily transmitted by the green leaf hopper (GLH; *Nephotettix virescens* (Distant)), while the transfer of the latter is RTSV dependent^12,14,15^. It has been reported that RTBV causes the tungro disease and RTSV intensifies the disease, hence most of the research focuses on RTSV resistance^16^. RT virus infection of rice plants results in stunted growth, leaf discoloration, delayed flowering and maturity, reduction in reproductive tillers, incomplete panicle exertion, improper grain filling and sterility leading to significant yield losses^13,17^. There have been multiple tungro mitigating measures such as use of chemicals, cultural and agronomic management practices, integrated tungro management approaches along with the use of resistant rice varieties^12,18^. However, the use of RT resistant rice varieties and the adoption of good agronomic practices are cost-effective, sustainable and environmentally safer^18^.

In the past, breeding for RT resistance was hampered mainly due to the confusion of whether to breed for resistance against vector carrier GLH or the RT virus^8,18,19^. But it has been well proven that breeding for RTSV resistance and deployment of RT resistant rice varieties is a practical approach to tackle RT disease in the farmers’ fields^17–21^. With the availability of high throughput RT virus screening phenotypic protocols coupled with enzyme linked immunosorbent assay (ELISA), it is possible to accurately identify resistance sources and to breed for tungro resistant rice varieties^22^. RT disease screening of several thousands of rice germplasm including gene bank accessions, breeding lines and released varieties, resulted in identification of many resistant donors for RTSV and only a few donors tolerant to RTBV^13,23–28^. Some of the prominent donors are Utri Merah, Utri Rajapan, Vikramarya ARC11554, IRGC16680, IRGC166682, Kataribhog, Pankhari 203 and many more. These accessions have been widely used in the genetic analyses and breeding for RT resistance in rice.

Identifying QTLs/genes for RT resistance helps in their marker-assisted introgression into popular rice varieties^8,13,28–31^. A dominant locus was identified on chromosome 4 that provided resistance to GLH and RTSV, which was later independently validated and fine mapped in a NSICRc138 and ARC138-4-5-5-2-30 derived back cross population^19,32^. While, several studies reported recessive genes contributing to RTSV resistance^29,33,34^. Similarly, QTLs were detected on chromosomes 1, 2 and 7 derived from Utri Rajapan and Vikramarya, each of these QTLs explained more than 16% phenotypic variance ^35^. A major effect loci “*tsv1”* for RT resistance was mapped between 22.05 and 22.25 Mb on the long arm of chromosome 7 using Utri Merah backcross derived population^8,21^. A putative translation initiation factor 4G (eIF4G^*tsv1*^) was found to be associated with *tsv1* for RTSV resistance, finally, an allele specific SNP was designed for use in MABB^8,36^. Recently, novel alleles of rice *eIF4G* were generated by CRISPR/Cas9 genome editing that conferred resistance to RTSV^37^. Some successful attempts were also made to develop RTBV resistance in the background of Pusa Basmati 1 through genetic engineering^20,37,38^.

Studies have clearly shown that most semi-dwarf green revolution rice varieties are susceptible to RT disease^39^. One of the classic examples is that the popular rice mega variety IR64, despite its wider adaptability, high yield potential, good grain quality traits and moderate to high level of resistance to most of the diseases, is highly susceptible to tungro disease^8,27,40,41^. In a phenotypic and molecular characterization of a set of rice lines along with commercially released rice varieties in Bangladesh, most rice varieties such as BR11, BRRI dhan39, BRRI dhan48, BRRI dhan49 and BRRI dhan71 were susceptible to RT disease^13^. Therefore, increasing rice crop resilience to sustain rice production is an important research priority^42^. Introgression or pyramiding of resistance genes into susceptible rice varieties using tightly linked diagnostic markers through marker assisted back cross breeding (MABB) is a fast track approach to control major diseases^43–46^. Even though QTLs/genes have been identified for RT resistance, there are not many efforts to systematically introgress them into popular rice varieties. For the first time, *tsv1* gene has been successfully introgressed into a *japonica* rice variety through MABB^21^.

Here we report the introgression of *tsv1* gene into BRRI dhan71, a popular drought tolerant rice variety of Bangladesh mainly grown during Aman season (wet season), it is widely grown in the northern and central parts of Bangladesh, where the farmers used to cultivate another crop between Aman and Boro seasons. It has a yield potential of 3.0–3.5 t/ha under drought and 4.0–5.0 t/ha under irrigated conditions^47^. But this variety has become highly susceptible to RT disease^13,48^.

The main objectives of our study were to conduct marker assisted introgression of *tsv1* into a popular rice variety of Bangladesh, BRRI dhan71; to characterize introgressed lines for agronomic traits, yield and RT resistance; to identify introgressed lines which were genotypically and phenotypically similar to recipient parent, and to conduct multi-location trials to identify superior lines for varietal nomination in Bangladesh.

## Results

### Selection of parental materials and *tsv1* linked SSR markers

The popular drought tolerant but RT susceptible rice variety BRRI dhan71 from Bangladesh was used as a recipient parent, for which the source of pure seeds came from Bangladesh Rice Research Institute (BRRI), Gazipur, Bangladesh. The well characterized RT resistant Matatag 1 was used as a donor parent. Both the parents grew well with good agronomic performance and yield potential at International Rice Research Institute (IRRI), Los Banos, Philippines. They are medium duration, BRRI dhan71 and Matatag 1 flowered 85 and 96 days after seeding, respectively, which helped in coinciding the flowering for making crosses. The polymorphism survey between the parents using eight simple sequence repeats (SSR) markers tightly linked to *tsv1* gene viz; RM336, RM21797, RM21800, RM6152, RM21801, RM21808, RM21822 and RM455, was carried out, of which two SSR markers RM336 and RM21801 were found to be polymorphic. They are located between 21.87 and 22.05 Mb region, RM336 is 180 kb away from *tsv1*, whereas, RM21801 marker is very tightly linked. These two markers were utilized for introgression of RT resistance gene into BRRI dhan71.

### Development of *tsv1* introgression lines through MABB

**Figure 1:**
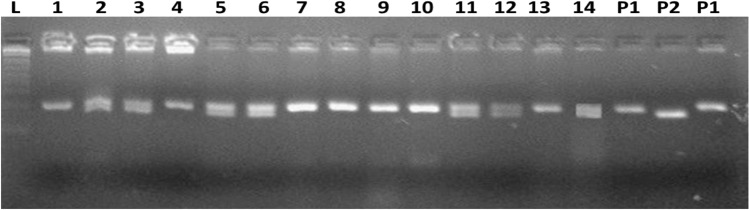
Gel picture showing RM336 maker segregation in BC_3_F_2_ generation. L—Ladder, P1—Susceptible (BRRI dhan71) and P2—Resistant (Matatag 1).

**Figure 2:**
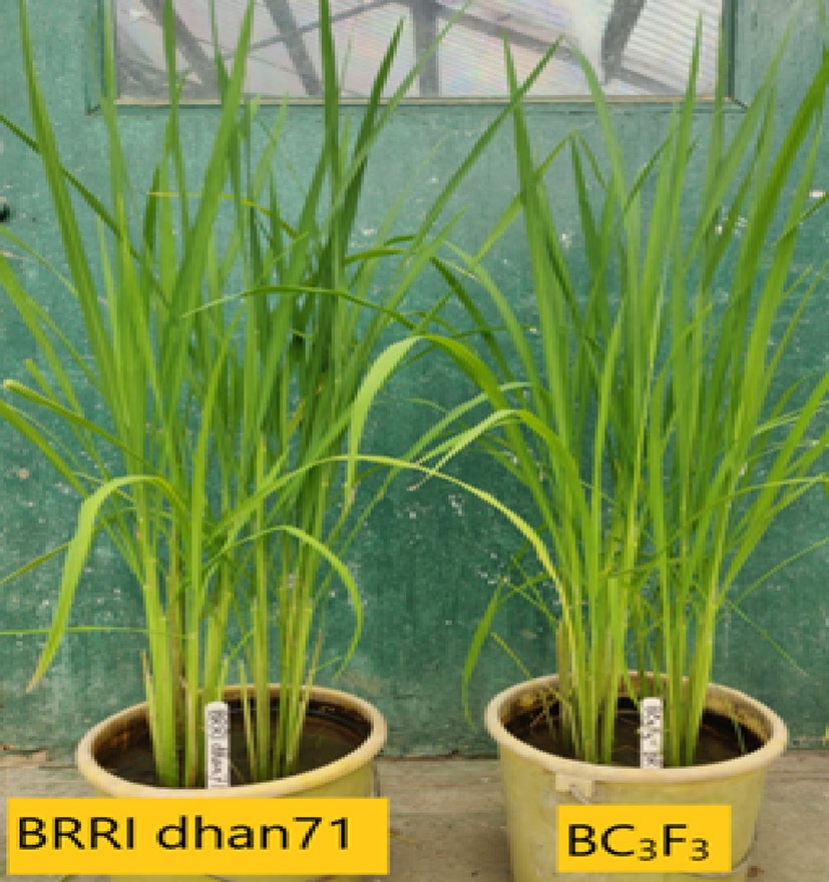
Introgression line with *tsv1* gene and recipient parent BRRI dhan71.

A cross was made using BRRI dhan71 as a female and Matatag 1 as a male parent. Both the parents were cross compatible and successfully produced F_1_s. The number of seeds produced, genotyped, true F_1_s and number of plants selected after each cross are provided in Table 1. We genotyped 157 out of the 223 F_1_ seeds produced, of which 83 were found to be true F_1_s when tested using SSR markers RM336 and RM21801. Eighteen positive plants were selected and backcrossed to BRRI dhan71 to produce BC_1_. In each of the backcross generations number of F_1_s produced and genotyped varied from 136 to 222 and 83–175, respectively, whereas the number of true F_1_s and those selected for crosses ranged from 43 to 98 and 14–27, respectively. From the 98 BC_3_F_1_ plants, 27 plants were selected similar to the recipient parent for plant type and grain type, and selfed them to produce BC_3_F_2_ plants. We pooled 10–15 seeds from each of the 27 plants to generate BC_3_F_2_ plants. In all 182 BC_3_F_2_ plants were genotyped out of which 37 were found to be homozygous for *tsv1* gene (Fig. 1). The DF and PH varied 75–98 days and 105–137 cm, respectively. Twenty three plants were selected based on agro-morphological characters resembling the recurrent parent and advanced them to obtain BC_3_F_3_ families. The markers segregation analysis was carried out and goodness of fit was applied for each of the back cross and BC_3_F_2_ selfing generation. In each of the back cross generations the segregation of markers followed 1:1 and in BC_3_F_2_ it was 1:2:1 (Table 2). Both the markers showed clear Mendelian inheritance without any segregation distortion. Overall, three successive MABB followed by selfing resulted in the identification of plants similar to the recurrent parent (Fig. 2).

**Table 1:** Number of seeds produced, plants genotyped and selected in back cross generations.

Generation	No. of seeds produced	No. of plants genotyped	True F_1s_	No. of plants selected
F_1_	223	157	83	18
BC_1_	153	95	46	14
BC_2_	136	83	43	26
BC_3_	222	175	98	27

**Table 2:** Segregation analyses of *tsv 1* linked markers in back cross generations.

Gen	No. of plants genotyped		No. of plants heterozygous		No. of plants homozygous		χ^2^-value		p- value	
	RM336	RM21801	RM336	RM21801	RM336	RM21801	RM336	RM21801	RM336	RM21801
BC_1_	95	95	46	49	49	46	0.095	0.095	0.758	0.758
BC_2_	83	83	43	45	40	38	0.108	0.591	0.742	0.442
BC_3_	175	175	98	100	77	75	2.52	3.571	0.112	0.058
BC_3_F_2_	182	182	88	92	38 + 56^a^	37 + 53^a^	3.758	2.835	0.152	0.242

^a^38 and 37 plants homozygous for *tsv1* gene, 56 and 53 plants homozygous for recipient allele in BC_3_F_2_ .

### Phenotypic and molecular characterization of introgression lines at IRRI-HQ

#### Agronomic, yield and yield related traits

**Figure 3:**
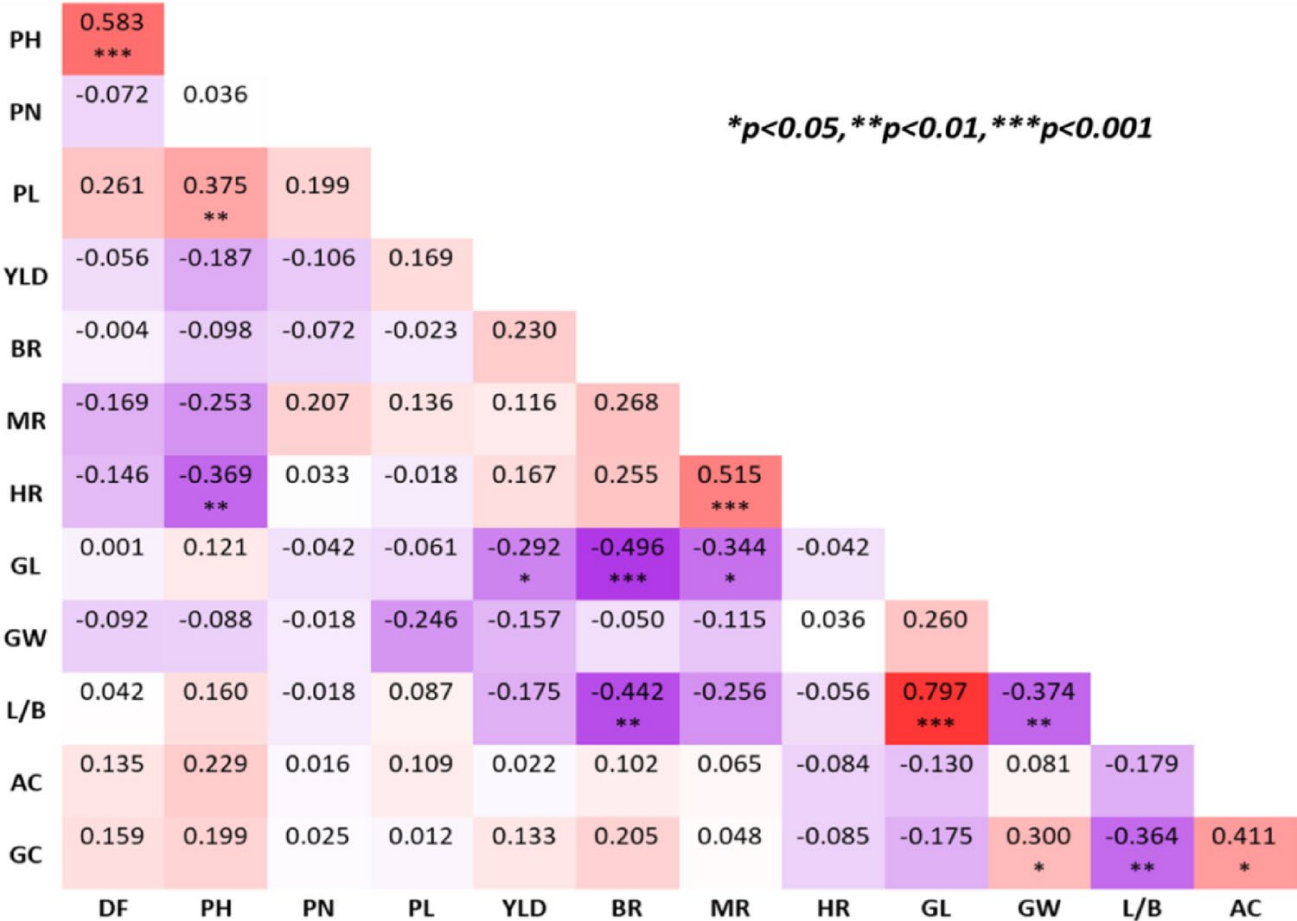
Correlation among agronomic, yield and grain quality related traits. DF—days to flowering (days), PH—plant height (cm), PN—panicle number, PL—panicle length (cm), YLD—yield (t/ha), BR-brown rice (%), MR-milled rice (%), HR—head rice recovery (%), GL—grain length (mm), GW-grain width (mm), L/B—length and breadth ratio, AC—amylose content (%), GC—gel consistency (mm).

Twenty three (BC_3_F_3_) and 50 ILs (BC_3_F_4_) were evaluated in replicated trials during 2021 dry season (DS) and 2021 wet season (WS), respectively. Among the ILs, range and means of DF, PN, PL and YLD were higher in DS, while PH was high in WS. Low to moderate coefficient of variations (CVs) was observed for all the traits during both seasons. There were significant genotypic differences for DF and YLD during both seasons, while PH has significant difference in WS but not in DS. PL has no significant genotypic difference in both seasons, whereas significant genotypic differences were observed for disease resistance reactions. For all the traits, moderate to high heritability (> 53%) were observed in both seasons (Table 3). The DF of BRRI dhan71 was 85 days and Matatag 1 had 96 days. BRRI dhan71 average PH was 121 cm. whereas; PL of BRRI dhan71 exhibited 27 cm with no significant difference. BRRI dhan71 exhibited an average yield of 4.2 ton/ha. Transgressive segregants were also observed for most of the traits. Overall, 62% of the ILs flowered earlier than BRRI dhan71; 14% and 50% of the ILs had longer panicle length and higher yield respectively, than BRRI dhan71. Among the agronomic traits, a significant positive correlation was observed between DF and PH (0.583), PH and PL (0.375), and a significant negative correlation was observed between GL and YLD (-0.292) (Fig. 3).

**Table 3:** Evaluation of BRRI dhan71 ILs for agronomic, yield and RTV resistance at IRRI-HQ.

Trait	Matatag 1	BRRI dhan71	Range	Mean ± SE	SD	CV (%)	H^2^ (%)	F-value	P-value	Season
DF	99.0	86.0	80–93	86 ± 0.49	3.5	4.1	67	4.4***	0.000	2021DS
	95.0	85.0	81–89	84 ± 0.20	2.5	2.9	92	11.8***	0.000	2021WS
PH	116.0	121.0	117–120	115 ± 0.85	6.0	5.2	79	1.9 ns	0.062	2021DS
	123.9	121.6	114–124	120 ± 0.48	5.9	5.0	81	1.8**	0.005	2021WS
PN	12.0	11.0	10–12	11.3 ± 0.28	2.0	17.7	55	1.4 ns	0.192	2021DS
	9.0	9.0	9–10	9.0 ± 0.08	1.0	11.3	67	1.2 ns	0.202	2021WS
PL	24.8	27.2	26–29	27 ± 0.21	1.4	5.2	69	1.18 ns	0.336	2021DS
	27.4	27.1	26–28	27 ± 0.07	0.9	3.3	93	0.9 ns	0.627	2021WS
YLD	6.2	6.6	3.9–7.8	6.1 ± 0.17	1.2	20.0	53	1.63*	0.041	2021DS
	2.5	4.2	3.6–4.9	4.2 ± 0.06	0.6	14.6	73	3.4***	0.000	2021WS
DI	3	6	3.0–5.0	3.9 ± 0.06	0.7	17.1	84	7.0***	0.000	2021WS

DF—days to flowering (days), PH—plant height (cm), PN—panicle number, PL—panicle length (cm), YLD—yield (t/ha), DI—disease index, SE—standard error, SD—standard deviation, CV—coefficient of variation (%), H^2^—heritability, * P≤ 0.05, ** P≤ 0.01, *** P≤ 0.001.

#### Grain quality assessment

Both the parents were similar for most of the grain quality traits except for HR and with slight differences in GL and LB ratio. Matatag 1 is a long slender grain type with LB ratio of 3 and BRRI dhan71 is a medium slender type with LB ratio of 2.5 (Table 4). Most of the ILs had medium slender grain type with a LB ratio of 2.63 and they were similar to BRRI dhan71. All the ILs, including the parents showed intermediate GT. The BR, MR and HR ranged between 73 and 80%, 64–73% and 58–65%, respectively, whereas BRRI dhan71 exhibited BR, MR and HR with 76%, 68% and 61%, respectively. The majority of ILs, showed 75%, 67% and 60% of BR, MR and HR, respectively. Low to moderate CVs were observed for all the traits among the ILs, and there were significant genotypic differences for GL and LB ratio. Among the grain quality traits significant positive correlations were observed between LB and GL with 0.797 followed by HR and MR (0.515), GC and AC (0.411), and GC and GW (0.300). A significant negative correlation was observed between GL and BR with − 0.496, followed by LB and BR (− 0.442), LB and GW (− 0.374), GC and LB (− 0.364), GL and MR (− 0.344) and GL and YLD (− 0.292) (Fig. 3).

**Table 4:** Variation for grain quality traits in the ILs.

Trait	Matatag 1	BRRI dhan71	Range	Mean ± SE	SD	CV (%)	t-value	P-value
BR	76.5	75.9	73.2–80.6	75.3 ± 0.16	1.2	1.5	− 3.68	0.999
MR	65.2	67.7	63.9–73.1	66.9 ± 0.23	1.6	2.5	− 3.39	0.999
HR	–	60.8	57.6–64.9	59.2 ± 0.87	6.3	10.5	− 2.25	0.985
GL	6.67	6.25	5.8–6.6	6.4 ± 0.02	0.1	2.1	8.65	0.000
GW	2.22	2.45	2.3–2.5	2.4 ± 0.01	0.1	1.8	− 1.33	0.188
L/B	3.00	2.55	2.4–2.8	2.63 ± 0.01	0.1	2.9	9.23	0.000
AC	19.8	20.1	18.9–21.2	20.1 ± 0.07	0.5	2.6	1.18	0.121
GC	42	47	29.0–60.0	40.1 ± 1.07	7.7	19.1	− 6.35	0.999
GT	I	I	I	I	–	–	–	–

BR—brown rice (%), MR—milled rice (%), HR—head rice recovery (%), GL—grain length (mm), GW—grain width (mm), L/B—length and breadth ratio, AC—amylose content (%), GC—gel consistency (mm), SE—standard error, SD—standard deviation, CV—coefficient of variation (%).

#### RT resistance screening

**Figure 4:**
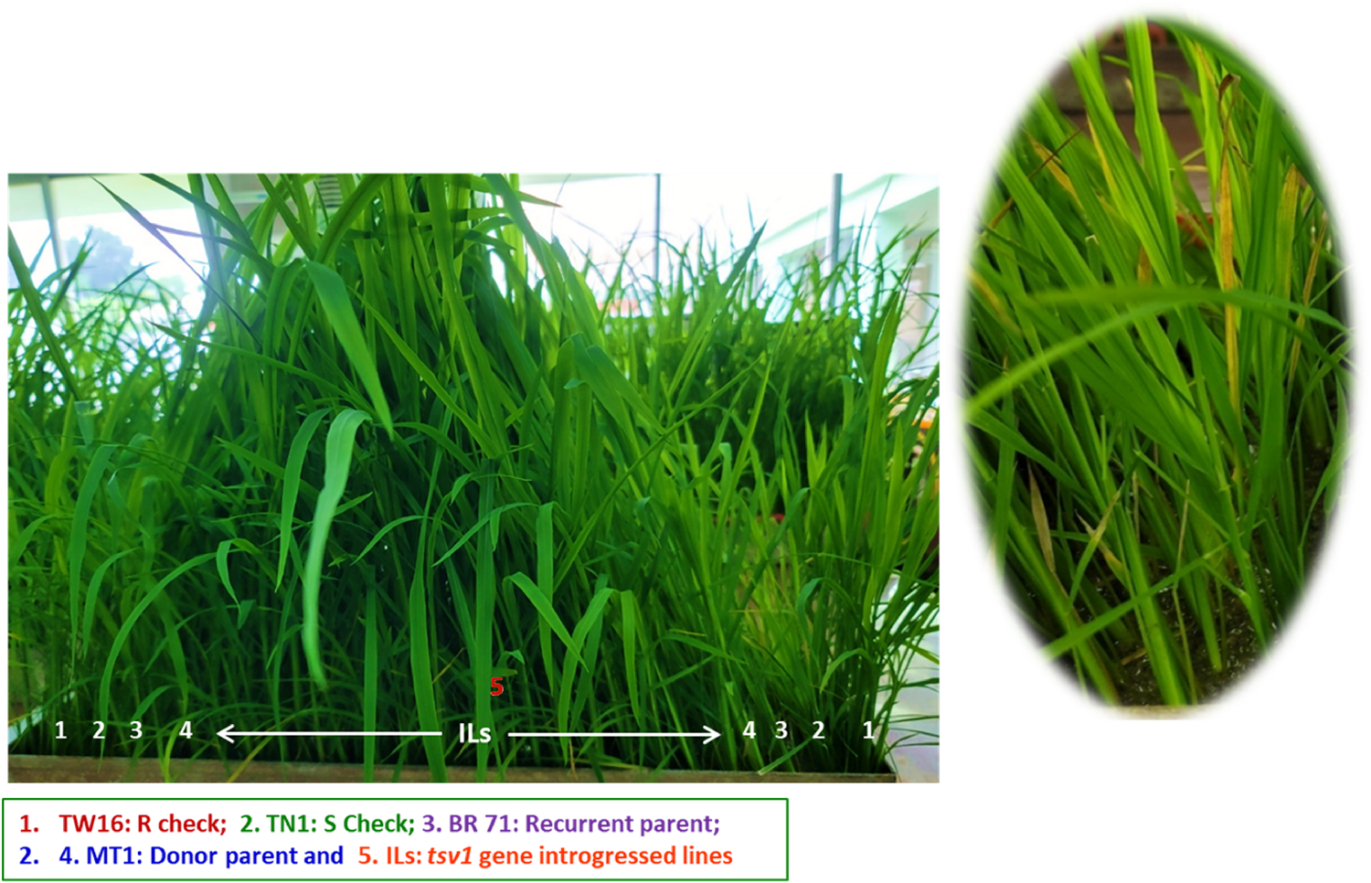
Plants are exhibiting symptoms of RT disease after inoculation. ILs are exhibiting resistant reaction against RT disease.

The RT disease reactions of the selected 50 ILs evaluated at IRRI-HQ is provided in the Table S1. As expected the donor parent Matatag 1 and the RT resistant check TW16 showed resistant reaction with a disease index (DI) score of 3, while the recipient parent and susceptible check TN1 showed susceptible reaction with DI score of 6 and 7. All the ILs performed better than recurrent parent BRRI dhan71 with a DI of 3–4 (Table 3 and Table S1). Seven ILs *viz*; IR 144469-1-1-1, IR 144469-1-1-6, IR 144481-1-2-3, IR 144483-1-2-6, IR 144485-1-1-1, IR 144485-1-1-3 and IR 144491-1-2-1 showed resistant reaction with DI of 3. All the selected ILs were healthy without any significant plant height reduction or yellowing of leaves even after 21 days of RT virus infestation (Fig. 4).

#### Background recovery

**Figure 5:**
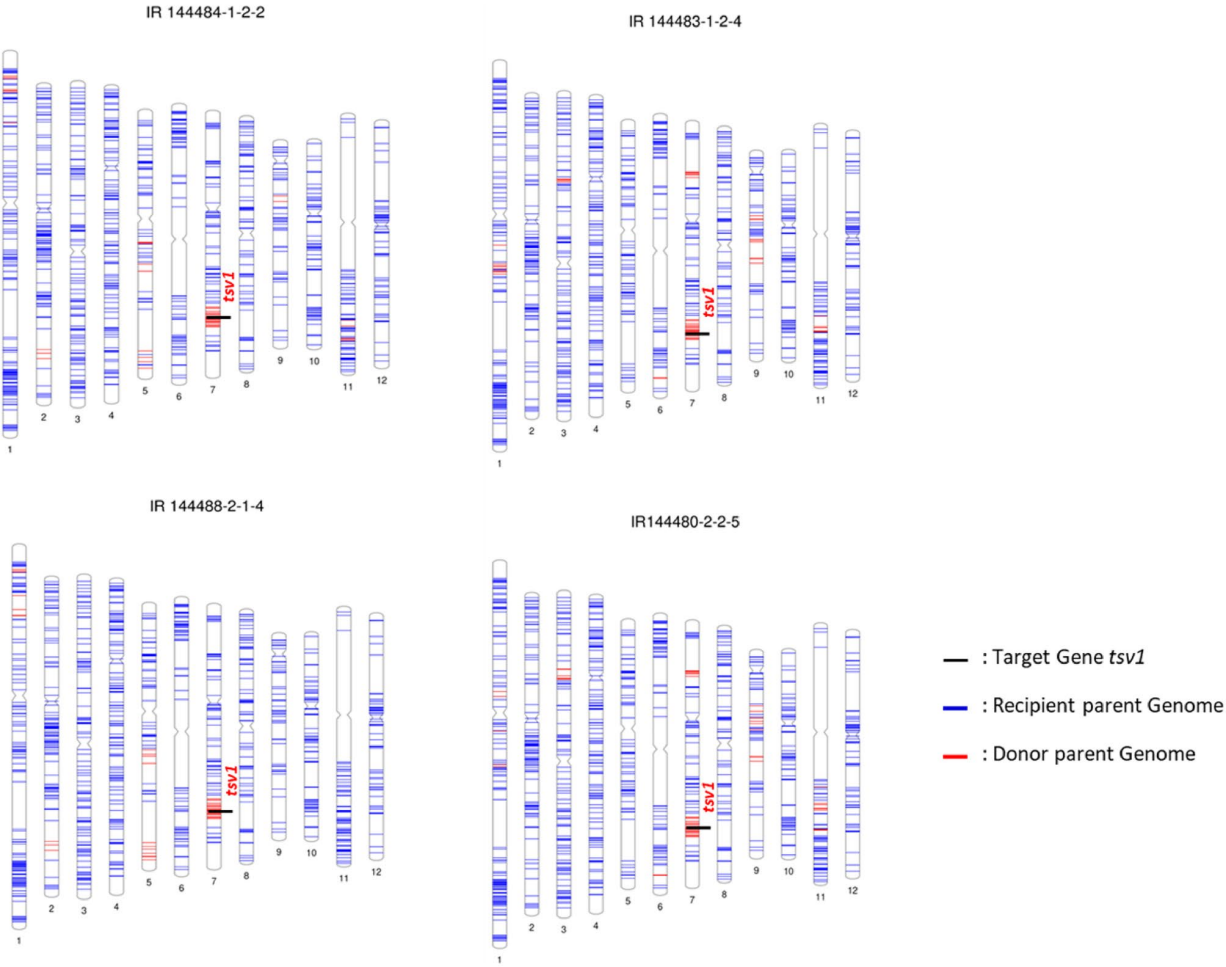
RPG recovery estimation of selected ILs.

Fifty ILs were subjected for 7 K SNP chip based genotyping, in all 7098 SNPs were successfully called. After filtering out missing (834), nonsense (195), heterozygous (14) and monomorphic (4994) SNPs, finally, 1061 high-quality polymorphic SNPs were extracted with an average marker density of one SNP per 300 kb. The polymorphic SNPs were distributed all over the genome. The number of polymorphic SNPs ranged from 57 on chromosome 9 to 148 on chromosome 1, with more than 100 markers on chromosomes 2, 3 and 4. The lowest numbers of donor allele introgressions were observed on chromosomes 4, 10 and 12 and highest on chromosome 7, on all other chromosomes, introgressions varied from 2.9 to 6.8%. The genetic background recovery of the 50 ILs showing alleles similar to the recipient parent varied from 91 to 95%. Overall in 38 ILs, background recovery was more than 95%. The graphical genotyping of the Recurrent Parent Genome (RPG) recovery of selected ILs showing the position of *tsv1* gene is provided in Fig. 5.

### Multi-location evaluation of introgression lines in Bangladesh

Agronomic performance of selected 50 ILs and their respective parents BRRI dhan71 and Matatag 1 were assessed at three RT disease hot spot locations during Aman season (WS 2021) in Bangladesh. We collected data for DF, PH, PN, PL and YLD in all three locations. There was no variation observed for DF among the lines and BRRI dhan71 across the locations, so it was excluded from the analyses. Results of individual location and combined analyses are provided in Table 5 and Table S2.

**Table 5:** Multi-location evaluation of BRRI dhan71 ILs in Bangladesh.

Trait	Loc	BRRI dhan71	Range	Mean ± SE	SD	CV (%)	H^2^ (%)	F-value	P-value
PH	GP	115	91–114	106 ± 0.57	7.2	6.8	70	3.3***	0.000
	CL	106	95–109	102 ± 0.32	4.0	3.9	89	8.9***	0.000
	HG	98	87–104	98 ± 0.38	4.8	4.9	68	1.9***	0.000
PN	GP	7	4–8	6 ± 0.10	1.3	20.2	61	2.5**	0.005
	CL	7	6–8	7 ± 0.09	1.2	16.6	46	1.1 ns	0.233
	HG	8	8–10	9 ± 0.13	1.7	18.1	61	2.5 ns	0.083
PL	GP	25	23.0–27.0	25 ± 0.11	1.3	5.3	57	2.3 ns	0.072
	CL	26	23.6–28.3	26.0 ± 0.14	1.7	6.7	45	1.1 ns	0.234
	HG	24	22.3–26.5	24 ± 0.10	1.3	5.4	64	2.7 ns	0.098
YLD	GP	6.5	2.8–6.9	5.1 ± 0.08	0.9	18.3	67	107.0***	0.000
	CL	4	2.1–4.8	3.4 ± 0.09	0.8	22.5	61	3.4***	0.000
	HG	6.1	2.5–7.9	4.5 ± 0.10	0.8	17.2	68	63.7***	0.000

PH—plant height (cm), PN—panicle number, PL—panicle length (cm), YLD—yield (t/ha), SE—standard error, SD—standard deviation, CV—coefficient of variation (%), H^2^—heritability, GP—Gazipur, CL—Cumilla, HG—Habiganj. ** P≤0.01, *** P≤0.001.

In the individual location analysis there were significant genotypic differences for PH and YLD among ILs in all the locations, while PL and PN among ILs and with BRRI dhan71 were almost similar. The mean PH (98 cm) was lowest at HG and the highest mean YLD (5.1 t/ha) was observed at GP. Low CVs were observed for PH (3.9–6.8%) and PL (5.3–6.7%), while moderate to high CVs were observed for PN (16.6–20.2%) and YLD (17.2–22.5%). Moderate to high heritability (> 45%) was observed for all the traits (Table 5).

**Figure 6:**
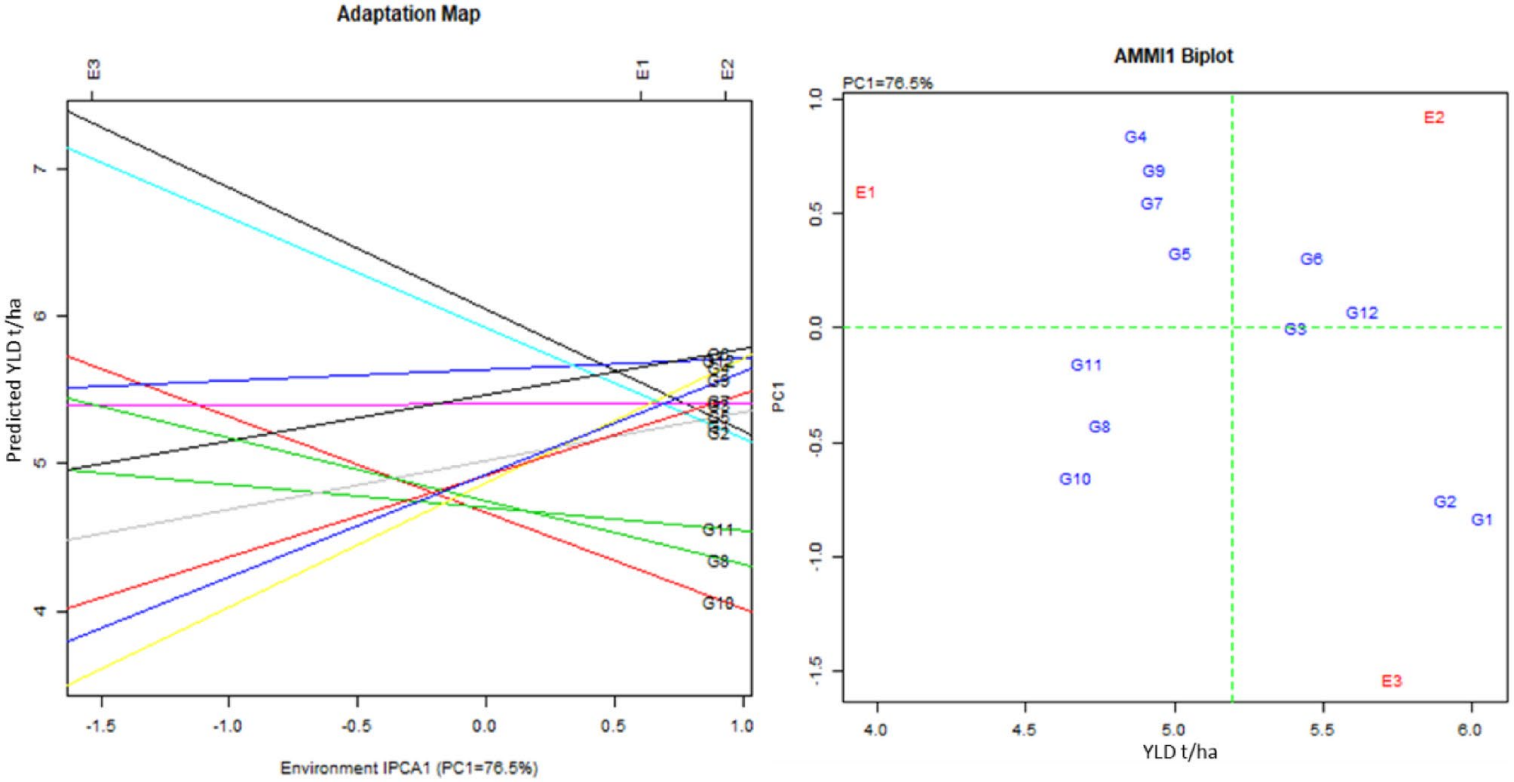
Adaption map and AMMI biplot. IR144480-2-2-5 (G1), IR144483-1-2-4 (G2), IR144484-1-2-2 (G3), IR144484-1-2-5 (G4), IR144485-1-1-6 (G5), IR144488-2-1-4 (G6), IR144489-1-1-1 (G7), IR144491-1-1-1 (G8), IR144491-1-2-5 (G9), IR144491-1-2-6 (G10), Matatag 1 (G11), BRRI dhan71 (G12), CL (E1), GP (E2) and HG (E3).

The AMMI analyses of variance showed significant genotypic effects for PH and YLD, environmental effects for PN and YLD, and significant genotype and environmental (G × E) interactions for PH and YLD. While among the first two PCs, PC1 was significant for all the traits but PC2 was significant for PN and YLD only (Table S2). AMMI biplot analysis showed 76.5% goodness of fit for YLD (Table S2 and Fig. 6) and revealed that the average yield was 5.2 t/ha. The two ILs G1 and G2, yielded higher than BRRI dhan71 with high main additive effects (Fig. 6), whereas the G3 and G6 yielded on par with BRRI dhan71 (G12). G3 and G6 are relatively close to PC1 zero score line, thus have wider adaptation to the test environments. The statistical analysis revealed some of the ILs viz., IR144480-2-2-5 (G1), IR144483-1-2-4 (G2), IR144484-1-2-2 (G3), IR144484-1-2-5 (G4), IR144485-1-1-6 (G5), IR144488-2-1-4 (G6), IR144489-1-1-1 (G7), IR144491-1-1-1 (G8), IR144491-1-2-5 (G9) and IR144491-1-2-6 (G10) performed stably in terms of yield across the three locations in Bangladesh (Table 6).

**Table 6:** Phenotypic performance and disease reaction of selected ILs evaluated at different locations.

Designation	GP			CL			HG			LB					
	DF	PH	YLD	DF	PH	YLD	DF	PH	YLD	DF	PH	YLD	DI	DR	RPG (%)
IR 144480-2-2-5 (G1)	90	105.4	6.6	88	103.1	3.5	92	96.4	7.9	85	122.4	3.9	4	M	95
IR 144483-1-2-4 (G2)	90	105.1	5.6	88	101.3	4.2	92	98.1	7.5	83	121.6	4.1	4	M	95
IR 144484-1-2-2 (G3)	90	106.1	6.3	88	104.7	3.8	92	98.0	6.0	86	122.3	4.1	4	M	95
IR 144484-1-2-5 (G4)	90	107.1	6.2	88	106.3	4.1	92	100.8	4.1	84	121.9	4.6	4	M	95
IR 144485-1-1-6 (G5)	90	108.1	5.3	88	103.8	4.4	92	99.9	4.9	85	122.8	3.7	4	M	95
IR 144488-2-1-4 (G6)	90	104.3	6.4	88	102.1	4.1	92	97.9	5.5	86	118.3	4.2	4	M	93
IR 144489-1-1-1 (G7)	90	103.4	6.1	88	102.5	3.8	92	97.2	4.6	85	119.3	4.6	4	M	93
IR 144491-1-1-1 (G8)	90	108.4	4.8	88	106.3	3.5	92	98	5.9	85	120.8	4.2	4	M	95
IR 144491-1-2-5 (G9)	90	108.6	6.8	88	105.8	3.5	92	98.3	4.5	83	114.8	4.1	4	M	91
IR 144491-1-2-6 (G10)	90	107.3	4.4	88	104.4	3.5	92	97.6	6.2	82	119.1	4.5	4	M	92
Matatag 1 (G11)	102	103.9	5.5	99	90.1	3.2	98	93.9	5.5	95	124.1	2.6	3	R	–
BRRI dhan71 (G12)	90	115.1	6.6	91	106.5	4.0	92	97.7	6.1	85	122	4.2	6	S	–

DF—days to flowering (days), PH—plant height (cm), YLD—yield (t/ha), DI—disease index, DR—disease reaction, LB—Los banos, GP—Gazipur, CL—Cumilla, HG—Habiganj, RPG—Recipient parent genome.

Stability analysis was performed for all the traits opting Kang’s stability model. The positive Ysi ranking indicates the stable performance of superior genotypes for different traits across the locations. Among the all, 50% of the ILs exhibited stable performance in at least one key yield trait for PN, PL and YLD and, 48% ILs showed stability for PH in all three locations (Table S3). The ILs IR144483-1-2-3, IR144485-1-1-1, IR144491-1-1-1, IR144491-1-2-3 and IR144492-1-3-2 were superior for PH, PN, PL and YLD traits; IR144480-2-2-5, IR144481-1-2-4, IR144485-1-3-1, IR144488-2-1-2 and IR144492-1-2-6 for PH, PN and YLD traits; and IR144482-1-1-1, IR144485-1-1-3, IR144491-1-2-1 and IR144491-1-2-5 for PH, PL and YLD traits. The Sum of YSi rankings indicates the stable performance of ILs across the locations. IR144491-1-1-1 was identified to have the highest YSi ranking (190) followed by IR144491-1-2-3 (167) and IR144485-1-1-1 (161), while the lowest YSi ranking observed in IR144469-1-1-6 (27) followed by IR144471-2-3-6 (29) and IR144469-1-1-1 (47). Whereas, the selected ILs *viz*., G1 (136), G2 (132), G3 (119), G4 (123), G5 (140), G6 (145), G7 (119), G8 (190), G9 (143) and G10 (121) exhibited moderate to high YSi ranking with mean yield of 4.33 t/ha across the three locations (Table S3).

### Identification of promising introgression lines

Based on the overall performance of ILs for yield and other traits, with good grain quality traits and resistance to RTV, four best promising ILs were identified, namely: IR144480-2-2-5 (G1), IR144483-1-2-4 (G2), IR144484-1-2-2 (G3) and IR144488-2-1-4 (G6). Three of the four lines had genetic background recovery of 95%. The agronomic and yield parameters were better or at par with the recipient parent BRRI dhan71 (Table 6). All these ILs had medium slender grain type with intermediate AC, GT and GC, and HR, BR and MR were almost similar to the BRRI dhan71 indicating their suitability for Bangladesh consumer preferences (Table S4). However, the whole set of 50 ILs are being further evaluated over the same three locations to identify stable lines.

## Discussion

Rice production continuously needs to be improved to sustain food security^4,49^. The increasing biotic stress pressure including the high incidences of RT disease on rice production is a major concern in Asia^37,50^. The RT is becoming epidemic in Bangladesh and most of the popular rice varieties released in Bangladesh have become susceptible to the disease. Studies have shown 20–100% yield losses, with none of the varieties tested showing complete resistance in the RT disease hotspot location at Cumilla, Bangladesh^13,39^. Hence, there is an urgent need to incorporate RT resistance into popular rice varieties through MABB or breed newer rice varieties with RT resistance^13,18^. The current study targeted to improve tungro resistance of a popular rice variety BRRI dhan71 through marker-assisted introgression of RT resistant gene “*tsv1*”.

### Marker assisted introgression of tungro resistance locus *tsv1*

Marker-assisted introgression of disease resistance genes using tightly linked markers is a fast-track approach to develop disease resistant varieties^21,49^. The foreground and background selections using molecular markers improve the precision and efficiency of the breeding programs to select desirable plants^51^. The *tsv1* is a recessive locus conferring resistance to RTSV, so it is more practical and appropriate to use MABB to develop RT resistant rice varieties^21^. It has been reported that RM336 is linked to *tsv1* and can be used in MABB^8,21^. However, this marker is located 180 kb away from the gene and showed a low-level recombination of 1.7% with the gene^8^. Polymorphism of eight SSR markers around the *tsv1* locus was surveyed between the parents BRRI dhan71 and Matatag 1, and polymorphic markers RM21801 and RM336 were used in our introgression work. The RM21801 is located close to *tsv1* and showed precise co-segregation with the gene. Interestingly, the study also observed over the different generations 3–4% recombination frequency between RM336 and RM21801 or *tsv1*. Hence both the marker loci to select positive plants in every generation were used to ensure the introgression of *tsv1*. It is important to note that an elongation initiation factor (eIFG^tsv1^) has been identified as a candidate gene and allele specific SNPs have been identified for *tsv1*^36^. With the availability of 3 K genome sequence better haplotypes can be searched for *tsv1*^52^. Use of superior haplotypes and tightly linked gene specific markers can further improve the efficiency of MABB^53^.

As expected, each of the successive backcrosses followed by selfing resulted in the production of plants similar to the recipient parent BRRI dhan71. The seed set, seed germination, and plant growth were normal and markers segregation followed Mendelian inheritance in each generation. These results clearly showed that the *tsv1* gene was introgressed and the recipient parent phenotype was successfully recovered without any segregation distortion (Table 2). Similar observations were also made in two populations derived from TW6 showing Mendelian segregation for *tsv1* by phenotypic segregation and genotypic segregation based on the progeny test^8^. Segregation distortion and linkage drag associated with the genes/QTLs derived from distant species of cultivated rice and wild relatives are a major hindrance for their use in breeding^54,55^. The main advantage of backcross is to retain the most important traits of the recipient varieties, it has been established that 3–4 backcrosses are enough to recover most of the recipient parent genome and thereby most of its traits^43,44,49^. RPG recovery was tested using genome-wide SNPs only in selected and fixed BC_3_F_4_ lines. The genetic RPG recovery varied from 91 to 95%, 38 (76%) out of the 50 ILs having 95% as per the theoretical expectation^43,56,57^. The stringent selection of progenies resembling recipient parent for agronomic and grain type traits in every back cross generation, and final selection of fixed plants similar to recipient parent resulted in expected genetic background recovery. However, in some ILs more numbers of donor specific alleles were introgressed around the *tsv1* gene on chromosome 7 compared to other regions of the genome.

A thorough phenotypic and molecular characterization of a wide range of rice germplasm showed the RT susceptibility of modern rice varieties including rice mega variety IR64, while genetic mapping efforts lead to identification of major loci for RT disease resistance but there are not many efforts to introgress them to popular rice varieties^13,23,28,35,41,58^. The study is the second report on *tsv1* introgression through MAS. In an earlier study *tsv1* was successfully combined with photoperiod insensitivity into japonica rice. They were able to identify a set of RT disease resistant breeding lines with high yield and good grain quality traits^21^. Similarly, a transgene *ORFIV* introgression into popular rice varieties conferred RT resistance^59^. However, recognizing the importance of RT resistance for stable rice production in Asia, it has been included as an important component of product profiles, SNPs have also been designed and validated for RT disease resistance and included in 1 K-RiCA assay for routine use in rice breeding^60^.

### Performance of ILs for agronomic, grain quality traits and RT disease resistance

The ILs showed consistently higher mean values and ranges for all the traits except PH during DS than in WS. There were significant genotypic differences for DF and YLD during both seasons, whereas PL has no significant genotypic differences. For all the traits, moderate to high heritability (> 53%) was observed in both seasons (Table 3). These results are in consonance with earlier reports on trait variations, genotypic effects and heritability of ILs for disease resistance, drought and submergence tolerance, and nutritional traits^9,21,43,57,61,62^. Transgressive ILs with early flowering, longer panicles and higher yield than BRRI dhan71 were observed. Similar observations were also reported on ILs of BB and blast resistance with early flowering, reduced height and increase in number of grains and no changes in panicle length^63,64^. The appearance of transgressive segregrants in the progenies is due to the accumulation of positive alleles from both the parents^65,66^. The phenotypic variation present in the ILs provides an opportunity to select superior lines for agronomic and yield related traits along with high level of stress tolerance or nutritional value.

Most of the ILs had medium slender grain type with intermediate AC, GT and GC, while BR, MR and HR were within the acceptable range. It shows that careful selection of lines and MABB resulted in development of ILs with unaltered grain quality parameters and these were similar to the recipient parent BRRI dhan71. Grain quality traits play an important role in consumer acceptance of released rice varieties^67^.

The RT virus inoculation resulted in stunting and severe yellowing of leaves of susceptible check and BRRI dhan71 (Fig. 4). All the ILs and resistant check showed moderate to high level of resistance compared to BRRI dhan71 (Table 3 and Table S1). All the selected ILs were healthy without any significant plant height reduction or yellowing of leaves even after 21 days of RT virus infestation while susceptible check could not recover. This clearly shows the effectiveness of *tsv1* and its successful introgression into BRRI dhan71 through MABB. Efforts are being made for additional controlled screen house based RT disease screening and forced tube inoculation to select the final set of lines. The effectiveness of *tsv1* and its successful transfer through MAS and further validation by phenotyping have been previously reported^8,21^. Similarly, MABB has been used to transfer disease resistance genes of BB, blast, sheath blight, tungro, drought and submergence tolerance etc. in rice, and promising ILs have been successfully released for farmers cultivation^21,32,40,43–46,61,68–70^.

### Tungro resistant ILs with superior agronomic and grain quality traits were identified

Multi-location evaluations of ILs were conducted at RT disease hotspot locations in Bangladesh. Understanding the GxE and identification of stable performing lines is essential for the successful release and adoption of rice varieties^71–73^. Significant genotypic, environment, and G × E effects were observed for YLD. Based on the AMMI and Kang’s stability analyses, ten best performing lines were selected (Table 6). These lines had DF and PH similar to BRRI dhan71, and YLD at par or better than BRRI dhan71. They had moderate to high level of resistance and all the grain quality traits were within the acceptable range. Final set of RT disease resistant lines selected based on Multi-location data will be nominated for varietal testing in Bangladesh.

## Conclusion

RT disease is becoming epidemic in several countries of Asia and most of the popular cultivated rice varieties are susceptible to the disease. Breeding for RT disease resistance is an effective approach to tackle the disease. RT disease resistant locus *tsv1* was successfully introgressed into a popular but RT susceptible rice variety BRRI dhan71 through MABB. The final set of ILs recovered most of the genetic background of the recipient parent, and showed agronomic, yield and grain quality parameters similar to the BRRI dhan71.

## Materials and methods

### Plant materials

BRRI dhan71 is a mid-early duration rice variety with promising yields even under drought conditions in Bangladesh but it is highly susceptible to RT disease. Because of its mid-early crop duration farmers are opting for this variety to grow in between Aman (wet) and Boro (dry) season. BRRI dhan71 was imported from Bangladesh Rice Research Institute (BRRI), Gazipur, Bangladesh. Matatag 1 or IR69726-116-1-3 is a Philippine rice variety used as a donor parent for the tungro resistance *tsv1* gene*,* which exhibits moderate resistance against RTSV^13,17^. The donor parent Matatag 1 is derived from a cross combination of IR52256-84-2-3/IR72//IR1561-228-3*2/Utri Merah (Acc 16682), and seeds were obtained from IRRI gene bank. This study complies with relevant institutional, national, international guidelines and legislation.

### Crossing program

**Figure 7:**
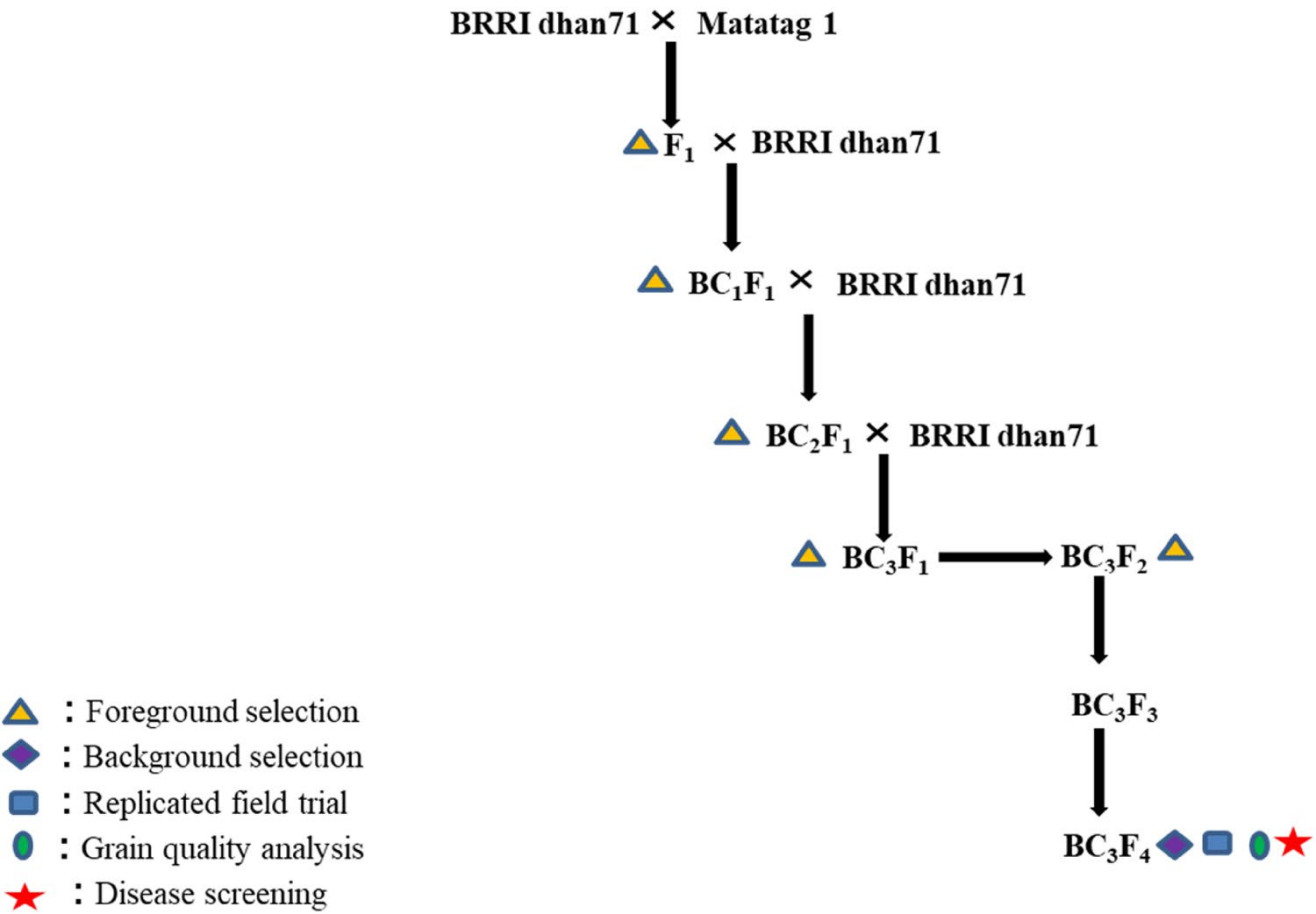
Breeding scheme for developing tungro resistant BRRI dhan71 ILs.

A standard MABB program to introgress *tsv1* from the donor parent Matatag 1 into BRRI dhan71 was followed. Crosses were made using Matatag 1 as a male parent and BRRI dhan71 was used as a female parent and F_1_s were produced. The F_1_s and BCnF_1_s produced were confirmed using *tsv1* gene tightly linked SSR markers RM336 and RM21801. The true F_1_ plants were backcrossed to the recipient parent three times and selfed to produce BC_3_F_2_ plants. The crossing scheme followed to develop BRRI dhan71 RTV resistance lines is provided in Fig. 7. In each of the generations, F_1_ plants with *tsv1* gene and phenotypically resembling recipient parent were selected and used for crosses. Selected BC_3_F_1_ was selfed to produce BC_3_F_2_ and *tsv1* homozygous plants were selected and further advanced through the pedigree method to produce BC_3_F_4_ (Fig. 7).

### Molecular analysis

#### DNA isolation and quantification

Young rice leaves (5–7 cm) were collected, lyophilized and each of the samples was grinded to powder in a 2 ml micro tube using GenoGrinder. Genomic DNA was extracted by TPS (a mixture of Tris–HCl, EDTA and KCl) method^74^. Pre-warmed 700 µl TPS buffer (100 mM Tris–HCl [pH 8.0], 1 M KCl, 10 mM EDTA) was added, well mixed and incubated at 65 °C for 1 h, and then centrifuged. The collected 500 µl supernatant was added with an equal volume of isopropyl alcohol and incubated at 4 °C for 30 min. Pellet was extracted after centrifugation and washed with 70% alcohol. The dried pellet was dissolved in 200 µl of TE buffer (10 mM Tris–HCl [pH 8.0], 1 mM EDTA, RNase A [10 mg/ml]). Quality and quantity of DNA were estimated through 0.8% agarose gel electrophoresis (AGE). We finally adjusted the DNA concentration to 25–50 ng/µl and used in the polymerase chain reaction (PCR). PCR using two SSR markers (RM336 and RM21801) tightly linked to *tsv1* gene was carried out ^8,13,21^. The primers sequences and product sizes are as follows.

RM336:FP-CTTACAGAGAAACGGCATCG; RP-GCTGGTTTGTTTCAGGTTCG; PS:154(bp); RM21801:FP-GCGCACAGCATGTCGAAGTCC; RP-AAACCCGAGGCAAATACGAAACG; PS:191(bp).

#### Polymerase chain reaction (PCR)

The PCR was performed using a total volume of 10 µl reaction mixture, containing 3 µl of 30 ng of template DNA and 0.5 µl of 5 Pico moles of each primer (Invitrogen), 1 µl of reaction buffer, 1 µl of dNTPs and 0.2 µl of *Taq* DNA polymerase. The PCR profile used was one cycle of initial denaturation at 95 °C for 5 min, followed by 35 cycles of amplification (each cycle of 1 min denaturation at 95 °C, 30 secs annealing at 55 °C, 30 secs extension at 72 °C), followed by a final extension of 72 °C for 7 min and stored at 4 °C. The amplified products were resolved in 3.5% Agarose gel with 0.5 X TBE buffer containing 0.1 μg/ml of Syber safe (S33102, Invitrogen) and documented in the Gel documentation system^21^. Gel scoring was carried out based on the donor and recurrent parent banding patterns and their corresponding product sizes. The allele corresponding with Matatag 1 was considered resistant allele. Similarly, allele matched with recipient parent BRRI dhan71 scored as a susceptible allele. Whereas if both the alleles (susceptible and resistant) were observed in one sample, they were considered heterozygotes.

### Phenotyping

#### Agronomic and yield parameters

Field experiments were conducted during 2021DS and 2021WS of 23 and 50 ILs, respectively at Zeigler experimental station (ZES), International Rice Research Institute. Randomized Complete Block Design (RCBD) was followed to lay out the experiments. Standard agronomic practices and plant protection measures to raise a good crop were followed. Data on agronomic and yield traits were collected following the standard evaluation system (SES of IRRI)^75^. Days to 50% flowering (DF) were calculated based on the sowing date to when 50% of the plants in a plot flowered. Plant Height (PH) was measured from the base of the plant to the tip of the tallest panicle from three selected plants; Panicle length (PL) was measured from the panicle base to the tip of the top most grain. After harvesting grains were well dried and moisture content was adjusted to 14%, grain weight per plot was measured in grams (gm) and converted to tons/ha.

#### Grain quality parameters

For grain quality (GQ) assessment, 140 g of well dried grains from each of replications were submitted at the Analytical Service Laboratory (ASL), IRRI. The grain morphological, milling and cooking quality data were estimated using standard protocols^76^. The GQ traits measured were grain length (GL), grain width (GW), brown rice (BR), milled rice (MR), head rice recovery (HR), amylose content (AC), gelatinization temperature (GT) and gel consistency (GC).

#### Disease screening for RT resistance

Parental lines and ILs for RT resistance were screened by artificial inoculation under controlled conditions. Materials were seeded in a metal tray following Randomized Complete Block Design (RCBD) with three replications under the controlled condition at disease screening facilities of IRRI. The Taichung Native 1 (TN1) and BRRI dhan71 were used as susceptible checks, while TW16 and Matatag 1 were used as resistant checks. Uniformity was maintained between entries in terms of the age of the seedlings and the number (with twenty seedlings) of seedlings per entry by thinning. Ten days old seedlings were subjected to RT virus infestation by releasing viruliferous GLH and allowed them to feed on seedlings. The insects were already given chances to feed on infected plants for 5 days. Seed box that contains the test seedlings were placed inside the water tray and covered with a screen. Viruliferous GLH was released in the cage at an average of 6–7 insects per seedling for 2–3 h of inoculation access. The insects were disturbed every 30 min during the inoculation period to ensure the even distribution among the seedlings. After 21 days of infestation, disease symptoms, incidence and severity were recorded (Fig. 4). Disease severity was evaluated based on a scoring system following SES^17,75^. Disease index (DI) was estimated based on disease percentage and symptoms. The DI value is determined by the severity of disease symptoms; the higher the DI value, the more severe symptoms^75^ (Table S5).

DI was estimated as follows

$$\text{Disease Index}= \frac{\text{No}.\text{ of plants showing Tungro symptoms}}{\text{Total No}.\text{ of plants observed}}\times 100$$

$$\text{Disease index}=\frac{\text{n}\left(1\right)+\text{n}\left(3\right)+\text{n}\left(5\right)+\text{n}\left(7\right)+\text{n}(9)}{\text{tn}}$$

where; n: the number of plants showed the corresponding score; tn*:* the total number of plants.

### Multi-location evaluation of introgression lines

Multi-location yield trials (MLTs) were conducted for selected 50 ILs at IRRI-HQ and at three tungro hot spot locations viz; Gazipur (GP), Cumilla (CL) and Habiganj (HG) in Bangladesh. The ILs along with their respective parents BRRI dhan71 and Matatag 1 were planted in three replications following a RCBD design and evaluated for agronomic traits, yield and disease reactions in the field conditions. Standard agronomic practices were adopted to raise healthier crops in all the field trials. Agronomic replicated data on DF, PH, PN, PL and YLD were collected as per the standard evaluation system of IRRI^75^.

### Statistical analyses

#### Phenotypic data analyses

All the basic statistical analyses were carried out using PB Tools v1.4 ^77^. The data were curated and means were estimated for each of the replications and used for the analyses. The descriptive statistical parameters such as range, mean, standard error (SE), standard deviation (SD), coefficient of variation (CV) and variance were estimated for each of the trials. Correlation analyses were performed by using R^78^. From the MLTs data Best Linear Unbiased Prediction (BLUPs) were estimated and used as input files to perform Additive Main-effects and Multiplicative Interaction (AMMI) biplot and genotype main effect analysis. Pairwise t-test of GQ data was performed using Statistical Tool for Agricultural Research (STAR) Version: 2.0.1.

Genotype means were estimated for each trial by using the following mixed model.

$$yijk = \mu + a_{i} + r_{j} + b_{lj} + e_{ijk}$$

here yijk is the performance of the i^th^ genotype in the k^th^ block of the j^th^ replication; μ represents the overall mean; *a*_*i*_ represents the effect of *i*^*th*^ genotype; *r *_*j*_ represents the effect of *j*^*th*^ replicate; *b*_*lj*_ the effect of *l*^*th*^ block within the *j*^*th*^ replicate; and *e*_ijk_ represents the random error.

Broad-sense heritability (H2) for each trait in each season was calculated as:

$${H}^{2}=\frac{{\sigma }_{g}^{2}}{{\sigma }_{p}^{2}} \text{and } {\sigma }_{p}^{2}= {\sigma }_{g}^{2}+\frac{{\sigma }_{e}^{2}}{r}$$

where ${\sigma }_{p}^{2}$ is the phenotypic variance, ${\sigma }_{g}^{2}$ is the genotypic variance, ${\sigma }_{e}^{2}$ is the error variance and r is the number of replications in the season.

#### Stability analysis

The effect of G × E interactions and performance of the genotypes in multi-locations trial in Bangladesh can be explained by stability models like Kang’s stability index. These models will enable to identify stable genotypes.

#### Genotypic data analyses

The segregation analysis of the *tsv1* gene was carried out using the marker segregation data of the RM336 and RM21801. We carried out Chi-square (χ^2^) analyses for the goodness of fit for each of the data sets from BC generations and BC_2_F_2_ using (Excel 2010). The RGP for each of the ILs was estimated based on the number of SNP alleles similar to the recipient parent to that of the total number of alleles amplified and expressed in percentage using Micro soft Excel.

## Supplementary Information

Supplementary Tables.

## Data Availability

The original contributions presented in the study are included in the article/Supplementary Material.
