# Supplementary material for: Introgression of tsv1 improves tungro disease resistance of a rice variety BRRI dhan71
Source: Sci Rep. 2022 Nov 5;12:18820. doi: 10.1038/s41598-022-23413-4 (PMC9637097; doi:10.1038/s41598-022-23413-4)
Supplement: Supplementary file 1 — Supplementary Tables. [file 41598_2022_23413_MOESM1_ESM.pdf]

Table S1: Results of disease index and reaction after RTV inoculation.

Designation	Disease Index	Disease Reaction
IR 144467-1-2-2	4	M
IR 144467-1-2-6	4	M
IR 144468-1-3-1	4	M
IR 144468-1-3-2	4	M
IR 144469-1-1-1	3	R
IR 144469-1-1-2	4	M
IR 144469-1-1-6	3	R
IR 144471-2-2-2	4	M
IR 144471-2-2-4	4	M
IR 144471-2-3-6	4	M
IR 144472-1-3-5	4	M
IR 144472-1-3-6	4	M
IR 144473-1-1-2	4	M
IR 144473-1-1-3	4	M
IR 144473-1-1-4	4	M
IR 144478-1-1-1	4	M
IR 144478-1-1-2	4	M
IR 144478-1-1-3	4	M
IR 144478-1-1-6	4	M
IR 144480-2-2-2	4	M
IR 144480-2-2-5	4	M
IR 144481-1-2-3	3	R
IR 144481-1-2-4	4	M
IR 144482-1-1-1	4	M
IR 144482-1-1-6	4	M
IR 144483-1-2-3	4	M
IR 144483-1-2-4	4	M
IR 144483-1-2-5	4	M
IR 144483-1-2-6	3	R
IR 144484-1-2-2	4	M
IR 144484-1-2-5	4	M
IR 144485-1-1-1	3	R
IR 144485-1-1-3	3	R
IR 144485-1-1-6	4	M
IR 144485-1-3-1	4	M
IR 144485-1-3-4	4	M
IR 144488-2-1-2	4	M
IR 144488-2-1-4	4	M
IR 144489-1-1-1	4	M
IR 144490-1-1-2	4	M
IR 144490-1-1-3	4	M
IR 144491-1-1-1	4	M
IR 144491-1-2-1	3	R
IR 144491-1-2-3	4	M
IR 144491-1-2-4	4	M
IR 144491-1-2-5	4	M
IR 144491-1-2-6	4	M
IR 144492-1-3-2	4	M
IR 144492-1-3-3	5	M
IR 144492-1-3-4	4	M
BRRI dhan71	6	S
TN1	7	S
Matatag 1	3	R
TW16	3	R

Table S2: AMMI analysis of variance for agronomic traits.

Source of variation	Multi-location evaluation of BRRI dhan 71 ILs in Bangladesh											
	PH			PN			PL			YLD		
	MS	P -value	VE%	MS	P- value	VE%	MS	P -value	VE%	MS	P -value	VE%
Gen	44.774	0.000	23.04	2.162	0.431	23.45	2.638	0.064	19.97	1.598	0.001	27.34
Env	2845.892	0.284	54.35	2013.756	0.030	65.73	1708.628	0.129	57.92	1616.909	0.000	68.12
Gen × Env	2845.892	0.000	21.23	2015.202	0.503	24.17	1940.642	0.423	21.23	1648.048	0.000	26.89
PC1	40.427	0.000	72.4	3.519	0.000	66.00	3.688	0.000	67.2	3.486	0.000	76.5
PC2	12.761	0.611	27.6	2.150	0.005	34.00	1.975	0.142	32.8	1.767	0.000	23.5

Gen- Genotype, Env- Environment, MS- Mean Square, VE%- variation explain

***Significant at $P<0.001$; **Significant at $P<0.01$; *Significant at $P<0.05$

Table S3. Stability index of ILs evaluated in MLT.

Designation	PH	PN	PL	YLD	YSi Sum
IR 144467-1-2-2	15	39+	25	13	92
IR 144467-1-2-6	8	9	11	34+	62
IR 144468-1-3-1	5	38+	28+	24	95
IR 144468-1-3-2	21	25	6	3	55
IR 144469-1-1-1	6	0	33+	8	47
IR 144469-1-1-2	2	3	2	14	21
IR 144469-1-1-6	12	8	1	6	27
IR 144471-2-2-2	0	2	14	35+	51
IR 144471-2-2-4	5	49+	8	22	84
IR 144471-2-3-6	20	-4	9	4	29
IR 144472-1-3-5	18	44+	3	-1	64
IR 144472-1-3-6	17	41+	20	1	79
IR 144473-1-1-2	10	50+	0	18	78
IR 144473-1-1-3	-7	1	5	21	20
IR 144473-1-1-4	3	37+	7	1	48
IR 144478-1-1-1	4	46+	44+	7	101
IR 144478-1-1-2	14	24	21	15	74
IR 144478-1-1-3	21	5	37+	2	65
IR 144478-1-1-6	28+	43+	42+	25	138
IR 144480-2-2-2	11	47+	30+	10	98
IR 144480-2-2-5	27+	51+	12	46+	136
IR 144481-1-2-3	13	28+	32+	19	92
IR 144481-1-2-4	32+	42+	18	28+	120
IR 144482-1-1-1	26+	36+	43+	1	106
IR 144482-1-1-6	8	22	47+	12	89
IR 144483-1-2-3	40+	34+	45+	36+	155
IR 144483-1-2-4	25	48+	10	49+	132
IR 144483-1-2-5	36+	45+	49+	11	141
IR 144483-1-2-6	34+	19	16	17	86
IR 144484-1-2-2	37+	16	17	49+	119
IR 144484-1-2-5	49+	7	23	44+	123
IR 144485-1-1-1	48+	30+	46+	37+	161
IR 144485-1-1-3	51+	14	52+	38+	155
IR 144485-1-1-6	42+	11	41+	46+	140
IR 144485-1-3-1	38+	35+	13	31+	117
IR 144485-1-3-4	39+	40+	40+	17	136
IR 144488-2-1-2	41+	32+	4	29+	106
IR 144488-2-1-4	24	23	48+	50+	145
IR 144489-1-1-1	19	10	45+	45+	119
IR 144490-1-1-2	22	6	36+	48+	112
IR 144490-1-1-3	29+	13	34+	14	90
IR 144491-1-1-1	47+	48+	53+	42+	190
IR 144491-1-2-1	52+	18	38+	31+	139
IR 144491-1-2-3	53+	31+	51+	32+	167
IR 144491-1-2-4	44+	20	31+	18	113
IR 144491-1-2-5	46+	17	33+	47+	143
IR 144491-1-2-6	35+	29+	19	38+	121
IR 144492-1-3-2	50+	33+	39+	31+	153
IR 144492-1-3-3	37+	10	48+	39+	134
IR 144492-1-3-4	23	21	22	26+	92
Matatag 1	-10	50+	24	43+	107
BRRI dhan71	47+	15	29+	52+	143
Trait Mean:	101.82	7.48	25.03	4.33	
YS Mean:	25.33	26.17	26.42	25.54	
LSD (0.05):	2.09	0.85	1.13	1.04	

Table S4: Grain quality traits of the selected ILs.

Designation	GL(mm)	GW(mm)	L/B Ratio	AC (%)	GT	GC(mm)	BR (%)	MR (%)	HR (%)
IR 144480-2-2-5	6.41	2.46	2.61	19.7	I	29	75.1	65.6	60.9
IR 144483-1-2-4	6.39	2.41	2.65	20.0	I	30	73.6	64.4	54.1
IR 144484-1-2-2	6.47	2.45	2.64	20.8	I	48	73.2	63.9	57.5
IR 144484-1-2-5	6.37	2.46	2.59	20.6	I	45	75.2	66.7	60.1
IR 144485-1-1-6	6.46	2.52	2.56	21.1	I	44	75.5	66.7	59.2
IR 144488-2-1-4	6.03	2.41	2.50	20.4	I	46	76.6	68.2	62.0
IR 144489-1-1-1	5.85	2.38	2.46	21.0	I	45	77.9	68.8	56.7
IR 144491-1-1-1	6.49	2.46	2.64	20.5	I	50	75.2	67.5	58.0
IR 144491-1-2-5	6.38	2.41	2.65	20.0	I	43	75.3	67.3	59.2
IR 144491-1-2-6	6.41	2.44	2.63	20.2	I	48	75.4	67.2	56.7
BRRI dhan71	6.25	2.45	2.55	20.1	I	47	75.9	67.7	60.8

Table S5: Disease severity scoring according to PH reduction and leaf discoloration.

Class	Plant height reduction (%)	Leaf discoloration	Disease Index	Remarks
1	0%	none	1	Highly resistant
2	1-10%	none	3	Resistant
3	11-30%	no distinct discoloration	5	Moderately resistant
4	31-50%	yellow to orange discoloration	7	Susceptible
5	above 50%	yellow to orange discoloration	9	Highly susceptible
